# The health-related quality of life among pre-diabetics and its association with body mass index and physical activity in a semi-urban community in Malaysia- a cross sectional study

**DOI:** 10.1186/1471-2458-14-298

**Published:** 2014-04-01

**Authors:** Norliza Ibrahim, Foong Ming Moy, Intan Attikah Nur Awalludin, Zainudin Ali, Ikram Shah Ismail

**Affiliations:** 1 Department of Social and Preventive Medicine, University of Malaya, 50603 Kuala Lumpur, Malaysia; 2 Julius Centre University of Malaya, Department of Social and Preventive Medicine, University of Malaya, 50603 Kuala Lumpur, Malaysia; 3 Diabetes Association, Petaling Jaya, 46200 Selangor, Malaysia; 4 State Health Department of Negeri Sembilan, Jalan Rasah, 70300 Seremban, Negeri Sembilan, Malaysia; 5 Department of Medicine, University Malaya Medical Centre, 50603 Kuala Lumpur, Malaysia

**Keywords:** Pre-diabetes, Health-related quality of life, Body mass index, Physical activity

## Abstract

**Background:**

People with pre-diabetes are at high risk of developing type 2 diabetes and cardiovascular diseases. Measurements of health-related quality of life (HRQOL) among pre-diabetics enable the health care providers to understand their overall health status and planning of interventions to prevent type 2 diabetes. Therefore we aimed to determine the HRQOL and physical activity level; and its association with Body Mass Index (BMI) among pre-diabetics.

**Methods:**

This was a cross sectional study carried out in two primary care clinics in a semi-urban locality of Ampangan, Negeri Sembilan, Malaysia. Data was collected through self-administered questionnaires assessing the demographic characteristics, medical history, lifestyle and physical activity. The Short Form 36-items health survey was used to measure HRQOL among the pre-diabetics. Data entry and analysis were performed using the SPSS version 19.

**Results:**

A total of 268 eligible pre-diabetics participated in this study. The prevalence of normal weight, overweight and obesity were 7.1%, 21.6% and 71.3% respectively. Their mean (SD) age was 52.5 (8.3) years and 64.2% were females. Among the obese pre-diabetics, 42.2% had both IFG and IGT, 47.0% had isolated IFG and 10.8% had isolated IGT, 36.2% had combination of hypertension, dyslipidemia and musculoskeletal diseases. More than 53.4% of the obese pre-diabetics had family history of diabetes, 15.7% were smokers and 60.8% were physically inactive with mean PA of < 600 MET-minutes/week. After adjusted for co-variants, Physical Component Summary (PCS) was significantly associated with BMI categories [F (2,262) = 11.73, p < 0.001] where pre-diabetics with normal weight and overweight had significantly higher PCS than those obese; normal vs obese [M_diff_ = 9.84, p = 0.006, 95% CI_diff_ = 2.28, 17.40] and between overweight vs obese [M_diff_ = 8.14, p < 0.001, 95% CI_diff_ = 3.46, 12.80].

**Conclusion:**

Pre-diabetics who were of normal weight reported higher HRQOL compared to those overweight and obese. These results suggest a potentially greater risk of poor HRQOL among pre-diabetics who were overweight and obese especially with regard to the physical health component. Promoting recommended amount of physical activity and weight control are particularly important interventions for pre-diabetics at the primary care level.

## Background

‘Pre-diabetes’ is defined as blood glucose concentrations higher than normal but not high enough to be classified as diabetes [1]. These conditions referred to patients who have either Impaired Fasting Glucose (IFG) and/ or Impaired Glucose Tolerance (IGT). More than 260 million people worldwide or 6.4% adults are estimated to have pre-diabetes [2]. People with pre-diabetes are at high risk of developing diabetes [3,4] and cardiovascular diseases [5,6]. Malaysia is a middle income country that is experiencing a rapid economic growth and urbanization in recent decades. This rapid transition has also led to an increase of pre-diabetes and diabetes prevalence in the country. According to Diabetes Atlas 2011, Malaysia ranked first among top ten countries with highest prevalence of IGT [2]. Furthermore, report from the National Health and Morbidity Survey (NHMS) 2011 showed the prevalence of diabetes in Malaysia rose from 11.6% (2006) to 15.2% (2011) in just 5 years. Approximately one out of five Malaysian adults suffer from diabetes. In addition to the above problems, the increasing prevalence of overweight and obesity in all segments of population has caused the burden of pre-diabetes and diabetes to continue to escalate [7]. Based on the national surveys in recent decades, the prevalence of overweight and obese adults in Malaysia increased from 17% and 4% respectively in 1996 to 29.4% and 15.1% in 2011 [8]. It is estimated that more than 8.5 million Malaysian adults are overweight and 4.4 million are obese with more than a quarter being physically inactive [9].

Health related quality of life (HRQOL) is defined as the overall impact of a medical condition on the physical, mental and social well being of an individual [10]. HRQOL measurements, including domains related to physical, mental, emotional and social functioning is a valuable health outcome [11]. This will help us to understand the patients’ overall health status, impact of treatment, formulation of health policy and decision on resource allocation [12]. Although there are many studies on relationship between HRQOL and Type 2 Diabetes [13-18], little is known about the relationship between HRQOL and pre-diabetes. Individuals in the general population who are overweight or obese and those with diabetes are significantly associated with impaired health-related quality of life [19-21]. In addition, overweight and obese adults who met the recommended level of physical activity had higher levels of HRQOL than physically inactive adults [22]. However, there is paucity of published studies of HRQOL among pre-diabetes patients in Malaysia. Therefore, in this study we aimed to determine the health-related quality of life among pre-diabetes patients and its association with the Body Mass Index (BMI). We also aimed to evaluate if differences existed in the HRQOL among pre-diabetes patients who were physically active (i.e. achieving ≥ 600 MET min per week) compared to those who were inactive within each BMI categories.

## Methods

### Study design and location

This was a cross sectional study carried out in two government primary care facilities located in a semi-urban locality of Ampangan, Negeri Sembilan in Malaysia. The Ampangan locality is about 70 km away to the south of Kuala Lumpur and covered an area of 89.12 km^2^ with estimated population of 130,823 people [23].

### Study procedures

**Figure 1:**
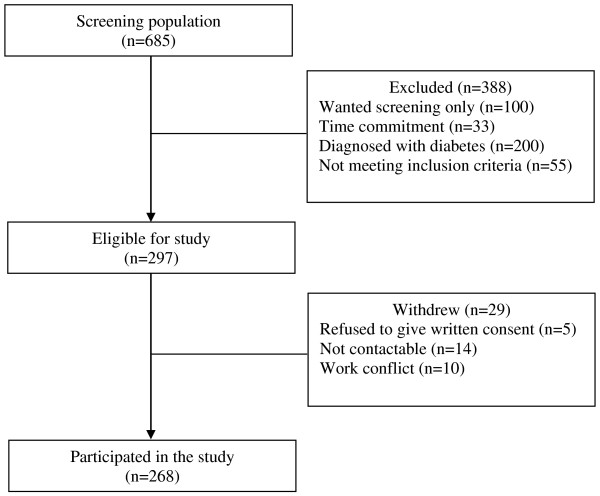
Diagram showing flow of participants through the study.

A total of 685 patients attending the two primary care clinics during the study period (between October, 2011 and March, 2012) were screened for eligibility to participate in the study. 388 were excluded because they wanted screening only, did not meet inclusion criteria, time work commitment and had a diagnosis of diabetes. 297 eligible patients aged 18 years old and above, were literate in Malay or English languages and who had been diagnosed with pre-diabetes by the physicians in the two primary care clinics were invited to participate. For this study, the diagnosis of pre-diabetes was based upon the American Diabetes Association (ADA) criteria in which Impaired Fasting Glucose (IFG) was defined as fasting plasma glucose concentration of between 5.6 to 6.9 mmol/L and Impaired Glucose Tolerance (IGT) was defined as fasting plasma glucose concentration of less than 7.0 mmol/L and a 2 hour postload plasma glucose concentration of between 7.8 mmol/L and 11.0 mml/L [24,25]. Patients with newly diagnosed diabetes, diagnosed with psychiatric illness or with any form of cognitive impairment such as mental retardation or dementia and females who were pregnant or breast feeding were also excluded. Prior to obtaining written consent, eligible participants were explained about the purpose of the study and relevant procedures involved. Finally, 268 patients who gave written consent were recruited in the study (Figure 1).

### Ethical issues

This study was approved by the Medical Ethics Committee of University of Malaya’s Medical Centre (MEC Ref. No. 841.3). Permission to conduct the study was also obtained from the State and District Health Directors as well as the Medical and Health Officers in charge of the selected clinics.

### Data collection and measurements

#### Socio-demographics and anthropometry measurements

All pre-diabetes patients attending the two primary health facilities were interviewed by trained medical staff using a set of structured questionnaires to obtain socio-demographic information, including age, gender, ethnicity, marital status, level of education, occupation and monthly household income. Patients were also asked about the presence or absence of co-morbid conditions such as hypertension and dyslipidemia, history of diabetes in the family, smoking and alcohol consumption.

The following anthropometric measurements were also taken. The standing heights of the patients without shoes were measured using a portable stadiometer (Seca, Vogel & Halke, Germany) and recorded to the nearest 0.1 cm. The weights of the patients with light clothing and without shoes were measured to the nearest 0.1 kg using a digital weighing scale (Seca Clara 803 Medical Scale, Germany). The Body Mass Index (BMI) was calculated using the formula of weight in kg divided by height in m^2^ (kg/m^2^). The current WHO classification states that the cut-off points for overweight and obesity BMI are ≥ 25 kg/m^2^ and ≥ 30 kg/m2 respectively [26]. However, it has become increasingly clear that there is a high prevalence of type 2 diabetes mellitus and cardiovascular risk factors occurring at BMI below 25 kg/m2 in Asian populations [27-29]. Many Asian populations have a higher body fat percent at similar BMI, compared with Caucasian/European populations [30-32]. In 2004, the World Health Organization (WHO) Expert Consultation revised the recommendation of BMI cut-offs for Asians [33]. Therefore, in this study we evaluate the associations between HRQOL and BMI according to the revised cut-offs for Asian classification. Patients with BMI between 18.5 kg/m^2^ to < 23.0 kg/m^2^ was classified as normal, a BMI between 23.0 kg/m^2^ to <27.5 kg/m^2^ was classified as overweight and a BMI of ≥ 27.5 kg/m^2^ was classified as obese [34].

#### Health and related quality of life (HRQOL) assessment

The HRQOL was assessed using a self-administered bilingual version of SF-36 health survey questionnaire. The SF-36 health survey questionnaire was translated and validated in Malaysia [35] and the Malay version of SF-36 has shown to be reliable and valid [36]. SF-36 has been widely used to compare quality of life of people with different diseases and those with chronic diseases [37] as well as among people who are disease free [15]. It contains 36 items which measures eight health concepts (domains): physical functioning (PF), role-physical (RF), bodily pain (BP), general health (GH), vitality (VT), social functioning (SF), role-emotion (RE) and mental health (MH) [38]. The eight domains were scored from 0 to 100 indicating worst to best possible health. All the scores were further summarized into the Physical Component Summary score (PCS) and the Mental Component Summary score (MCS).

#### Physical activity (PA) assessment

Physical activity (PA) was assessed using the short form International Physical Activity Questionnaire (IPAQ short form) [39]. The IPAQ was translated and validated in different languages including the Malay version of IPAQ [40]. This short version comprises seven items that identify frequency and time spent on three specific types of physical activity which are walking, moderate intensity activity and vigorous intensity activity during the past seven days. The Metabolic Equivalent (MET) values were used for measurements. The patients’ total physical activity MET-minutes/week was calculated by summing up the walking, moderate and vigorous intensity activity scores. Based on public health guidelines [41] and recommended thresholds [42], patients were categorized as “active” if they achieved ≥ 600 MET-minutes/week (equal to ≥150 minutes of moderate activity/week) and those achieved less were considered as “inactive”.

#### Data management and statistical analysis

All questionnaires were checked for completeness and attempts were made for missing items. Data were entered and analysed using Statistical Package for Social Science (SPSS, Inc, Chicago,IL) version 19.0. The descriptive analysis for socio-demographic, health and lifestyle characteristics were presented as means with standard deviation (SD) and frequency (percentage). Spearman rank correlation coefficient test was done to explore the magnitude and relationships between variables that potentially influenced HRQOL. To evaluate the influence of different BMI categories on the overall HRQOL scores, we performed analysis of variance (ANOVA) or its equivalent non-parametric Kruskal-Wallis test to compare the unadjusted means of SF-36 domain scores and BMI categories. Post-hoc analysis was used to analyze the mean score of each SF-36 domain among different BMI categories. For multivariate analysis, we performed general linear model of Multivariate Analysis of Covariance (MANCOVA) with HRQOL outcome scores (i.e.; Physical Component Summary and Mental Component Summary) as dependent variables, BMI categories as the independent variables while adjusting for co-variates. We also expanded our analysis to account for associations between HRQOL and PA within each of the BMI categories. Preliminary checks were conducted to ensure there were no violations of assumptions of normality, linearity, homogeneity of regression and reliable measurements of the covariates. We reported multivariate significance using Wilks’ λ statistic. Test of between-subject effects was conducted using Bonferroni adjustment to reduce the chance of type 1 error and we reported our results significant if the p-value is less than 0.025.

## Results

### Characteristics of study subjects

A total of 268 pre-diabetes patients were recruited in this study. The mean (SD) age of all patients was 52.5 (8.3) years. Majority of the pre-diabetes patients were females, Malays, married, completed secondary level of education and currently unemployed, retired or homemaker with low economic background. More than half of the pre-diabetes patients had family history of diabetes. The mean (SD) BMI of all pre-diabetes patients was 30.1 (4.8) kg/m^2^. When divided into BMI categories, 7.1% of them were of normal weight, 21.6% overweight and 71.3% obese. Among the obese group with pre-diabetes; 46.1% had both IFG and IGT, 44.5% had isolated IFG and 9.4% had isolated IGT. Those who were obese also had high prevalence for CVD risk factors; nearly half of them had hypertension and one third had a combination of hypertension, dyslipidemia and musculoskeletal illness. About 15.7% of patients were smokers and most were physically inactive with mean PA of < 600 MET-minutes/week (Table 1).

**Table 1:** Socio-demographic and health characteristics of pre-diabetes patients (n = 268)

**Characteristics**	**n (%)**
**Age in years, mean (SD)**	52.5 (8.3)
**Gender**	
Male	96 (35.8)
Female	172 (64.2)
**Ethnicity**	
Malay	223 (83.2)
Indian	23 (8.6)
Chinese	22 (8.2)
**Marital status**	
Unmarried/divorced/widowed	33 (12.3)
Married	235 (87.7)
**Education level**	
Primary	50 (18.7)
Secondary	174 (64.9)
Tertiary	44 (16.4)
**Employment status**	
Unemployed/retired/homemaker	145 (54.1)
Employed	123 (45.9)
**Monthly household income**	
Low (MYR <1500)	123 (45.9)
Moderate (MYR 1501-3500)	95 (35.4)
High (MYR > 3500)	50 (18.7)
**Family history of diabetes**	
No	125 (46.6)
Yes	143 (53.4)
**Diagnosis of prediabetes**	
IFG	126 (47.0)
IGT	29 (10.8)
Both	113 (42.2)
**Co-morbidities**	
No reported co-morbidities	32 (11.9)
Hypertension	126 (47.0)
Dyslipidemia	13 (4.9)
Hypertension, Dyslipidemia & Muskuloskeletal disease	97 (36.2)
**Body Mass Index (BMI)**	
BMI in kg/m^2^, mean (SD)	30.1(4.8)
Normal weight (18.5 – 22.9 kg/m^2^)	19 (7.1)
Overweight (23.0 - 27.4 kg/m^2^)	58 (21.6)
Obese (≥ 27.5 kg/m^2^)	191 (71.3)
**Smoking**	
No	226 (84.3)
Yes	42 (15.7)
**Alcohol**	
No	247 (92.2)
Yes	21 (7.8)
**Physical Activity (PA)**	
Total MET-minutes/week, mean (SD)	625.3 (480.8)
Non-active	163 (60.8)
Active	105 (39.2)

Notes: Data are presented as mean (standard deviation) for continuous variables and frequency (%) for categorical variables.

IFG: Impaired Fasting Glucose, IGT: Impaired Glucose Tolerance. MYR: Malaysian Ringgit. Classification from Department of Statistics Malaysia (currency conversion: 1.00 USD = MYR 3.07).

### The influence of Body Mass Index and Physical Activity on SF-36 scores

The influence of BMI on individual SF-36 scales of the HRQOL is presented in Table 2. The lowest mean score was General Health (M = 70.49) while the highest mean score was Social Functioning (M = 91.70). Before adjusting for selected socio-demographic variables known to influence HRQOL, pre-diabetes patients who were obese generally reported lower scores for most of SF-36 scales when compared to pre-diabetes patients with normal weight, indicating poorer HRQOL. Significant differences were found in the physical health component subscale scores; Physical Functioning (PF), Bodily Pain (BP), General Health (GH) and Physical Component Summary (PCS) except for Role Physical (RP). A post-hoc analysis showed significant differences were between the normal weight vs obese groups (p = <0.001) and overweight vs obese groups (p = <0.001). However, the mental health component subscale scores; Vitality (VT), Social Functioning (SF), Role Emotion (RE), Mental Health (MH) and Mental Component Summary (MCS) did not show any significant difference between BMI categories.

**Table 2:** Mean scores of SF-36 domains with different Body Mass Index categories

**SF-36 domains**	**BMI categories**					
	**Total, n=268**	**Normal, n=19**	**Overweight, n=58**	**Obese, n=191**	**F-stats**	**p-value**
	**Mean (SD)**	**Mean (SD)**	**Mean (SD)**	**Mean (SD)**		
PCS	81.03 (13.20)	88.01 (9.80)	86.78 (11.11)	78.59 (13.33)	12.23	<0.001*^a,b^
PF	81.92 (16.59)	84.47 (19.06)	87.41 (13.83)	79.99 (16.78)	4.82	0.009*^b^
RP	86.13 (19.20)	83.22 (23.39)	88.64 (20.52)	85.66 (18.36)	0.77	0.465
BP	84.35 (17.09)	92.16 (13.19)	94.19 (11.35)	80.59 (17.47)	18.31	<0.001*^a,b^
GH	70.49 (17.34)	74.68 (13.45)	76.88 (15.42)	68.14 (17.73)	6.51	0.002*^b^
MCS	83.85 (11.54)	85.52 (13.07)	85.55 (9.60)	83.16 (11.91)	1.17	0.313
VT	74.39 (13.36)	75.98 (12.88)	77.69 (12.83)	73.23 (13.45)	2.65	0.072
SF	91.70 (15.43)	92.76 (15.20)	93.53 (13.08)	91.04 (16.12)	0.62	0.537
RE	87.29 (18.58)	89.91 (19.94)	87.21 (18.15)	87.05 (18.65)	0.20	0.816
MH	82.01 (12.12)	83.42 (12.25)	83.79 (10.10)	81.34 (12.65)	1.05	0.351

Notes: One-way ANOVA or its equivalent Kruskal-Wallis test was used to compare the mean scores of each domains, *significant difference at p<0.05. Post-hoc analysis using Tamhane’s test. ^a^(significant difference between normal vs obese), ^b^ (significant difference between overweight vs obese).

BMI categories is based on WHO revised cut-offs for Asian: normal (18.5-22.9) kg/m^2^, overweight (23.0-27.4) kg/m^2^ and obese (≥ 27.5 ) kg/m^2^. PF: Physical Functioning, RP: Role limitations due to Physical health, BP: Bodily Pain, GH: General Health, VT: Vitality, SF: Social Functioning, RE: Role Emotions due to mental health, MH: Mental Health, PCS: Physical Component Summary and MCS: Mental Component Summary.

Correlations between HRQOL, socio-demographics and health characteristics of pre-diabetes patients are presented in Table 3. Spearman’s correlation coefficients (r) indicated that Physical Component Summary (PCS) was significantly correlated with BMI (r = −0.374, p < 0.01), physical activity (r = 0.123, p < 0.05) and income (r = 0.170, p < 0.01), whereas the Mental Component Summary (MCS) was significantly correlated with income (r = 0.148, p < 0.05). Variables with significant p value in the correlation analysis and thought to be important risk factors of HRQOL were entered into the multivariate model.

**Table 3:** Bivariate Spearman’s correlations between HRQOL, socio-demographic and health characteristics

**Variables**	**1**	**2**	**3**	**4**	**5**	**6**	**7**	**8**	**9**
**1. Age**	1.000								
**2. Gender**	−0.123^*^	1.000							
**3. Income**	−0.193^**^	−0.211^**^	1.000						
**4. Smoking**	−0.012	−0.513^**^	0.178^**^	1.000					
**5. No of co-morbidities**	0.181^**^	−0.066	0.009	0.044	1.000				
**6. BMI**	−0.269^**^	0.179^**^	0.003	−0.055	0.036	1.000			
**7. PA**	0.201^**^	0.034	−0.100	−0.080	0.021	−0.283^**^	1.000		
**8. PCS**	0.028	−0.107	0.170^**^	0.079	0.031	−0.374^**^	0.123^*^	1.000	
**9. MCS**	0.051	−0.024	0.148^*^	−0.006	0.055	−0.081	0.001	0.545^**^	1.000

Notes: significance correlation at *p < 0.05 level and **p < 0.01 level (2-tailed).

PA: Physical activity in total MET-minutes/week, BMI: Body mass index in kg/m^2^, No of co-morbidities range from 0 (i.e., no reported co-morbidities) to 3 (i.e., all 3 co-morbidities were present), PCS: Physical Component Summary scores, MCS: Mental Component Summary scores.

MANCOVA analysis was used to control the confounding effects of age, income, number of co-morbidities and physical activity level on the HRQOL when comparing between the BMI categories. After adjustment for the co-variants, the influence of BMI on SF-36 scores persisted [Wilk’s λ =0.90, F (4, 522) = 6.86, p < 0.001, partial eta square = 0.10, observed power = 0.99]. The BMI was significantly associated with PCS [F (2, 262) = 11.73, p < 0.001, partial eta square = 0.10, observed power = 0.99]. However, there was no significant association between MCS and BMI categories [F (2,262) = 0.98, p = 0.374, partial eta square = 0.007 and observed power = 0.22]. Difference in the means of PCS and MCS between normal, overweight and obese pre-diabetes patients is presented in Table 4. The obese pre-diabetes patients showed significant lower PCS scores than the normal weight (p = 0.006) and overweight patients (p < 0.001) respectively.

**Table 4:** The estimated means of HRQOL between normal, overweight and obese pre-diabetes patients adjusted for age, income, number of co-morbidities and physical activity

**SF-36**	**BMI**	**Adjusted mean (SE)**	**Between groups differences**				
			**BMI**	**M**_**diff**_	**SE**	**p-value**^**a**^	**95% CI**
PCS	Normal	88.42 (2.97)	Normal vs Overweight	1.70	3.37	1.000	−6.43, 9.83
	Overweight	86.72 (1.68)	Normal vs Obese	9.84	3.14	0.006*	2.28, 17.40
	Obese	78.57 (0.93)	Overweight vs Obese	8.14	1.93	<0.001*	3.46, 12.80
MCS	Normal	85.58 (2.71)	Normal vs Overweight	0.14	3.08	1.000	−7.57, 7.29
	Overweight	85.44 (1.54)	Normal vs Obese	2.38	2.86	1.000	−4.53, 9.29
	Obese	83.19 (0.85)	Overweight vs Obese	2.24	1.77	0.621	−2.03, 6.51

Note: Data were analyzed using MANCOVA test. ^a^Multiple comparisons using Bonferroni test. *The mean difference is significant at p<0.025 level. PCS = Physical Component Summary, MCS= Mental Component Summary, M_diff_ = Mean difference and SE= Standard Error, CI= Confidence Interval.

**Figure 2:**
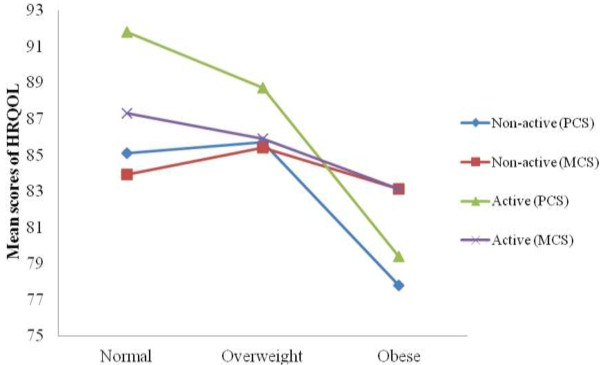
Mean score in Physical Component Summary (PCS) and Mental Component Summary (MCS) stratified by status of physical activity versus Body Mass Index (BMI) categories.

Table 5 displays the difference in estimated means of HRQOL stratified by PA across different BMI categories. Among the active patients, significant differences in the PCS scores were observed between normal weight vs obese and overweight vs obese patients (p = 0.007) and (p = 0.002) respectively. Similar significant difference in the PCS scores were also observed among the inactive patients between overweight and obese patients (p = 0.01). Figure 2 showed the overlapping of the PCS mean scores for both overweight and obese pre-diabetes patients who were physically active and inactive.

**Table 5:** The difference in estimated means of HRQOL stratified by Physical Activity status between normal, overweight and obese pre-diabetes patients

**SF-36**	**BMI**	**Adjusted mean (SE)**	**Between groups differences**				
			**BMI**	**M**_**diff**_	**SE**	**p-value**^**a**^	**95% CI**
**Non active**							
PCS	Normal	85.11 (4.41)	Normal vs Overweight	−0.61	4.98	1.000	−12.66, 11.44
	Overweight	85.71 (2.37)	Normal vs Obese	7.24	4.59	0.350	−3.86, 18.34
	Obese	77.87 (1.16)	Overweight vs Obese	7.85	2.65	0.011*	1.43, 14.27
MCS	Normal	83.94 (4.06)	Normal vs Overweight	−1.47	4.58	1.000	−12.57, 9.61
	Overweight	85.42 (2.18)	Normal vs Obese	0.82	4.22	1.000	−9.40, 11.04
	Obese	83.12 (1.07)	Overweight vs Obese	2.29	2.44	1.000	−3.62, 8.21
**Active**							
PCS	Normal	91.88 (3.66)	Normal vs Overweight	3.14	4.22	1.000	−7.13, 13.40
	Overweight	88.75 (2.17)	Normal vs Obese	12.47	3.96	0.007*	2.82, 22.12
	Obese	79.41 (1.41)	Overweight vs Obese	9.34	2.61	0.002*	2.97, 15.69
MCS	Normal	87.34 (3.52)	Normal vs Overweight	1.37	4.06	1.000	−8.51, 11.24
	Overweight	85.97 (2.09)	Normal vs Obese	4.26	3.81	0.800	−5.02, 13.54
	Obese	83.08 (1.35)	Overweight vs Obese	2.89	2.51	0.758	−3.23, 9.01

Note: Data were analyzed using MANCOVA test adjusted for age, income and number of co-morbidities. ^a^Multiple comparisons using Bonferroni test. *The mean difference is significant at p<0.025 level. PCS = Physical Component Summary, MCS= Mental Component Summary, M_diff_ = Mean difference and SE= Standard Error, CI= Confidence Interval.

## Discussion

The mean age and the age range of pre-diabetes patients in our study were similar to another study carried out previously among pre-diabetes Malaysian adults [43]. Interestingly, the prevalence of both IFG and IGT was 42.2% in our study. This condition increased the risk for diabetes as progression rates to diabetes were higher in people with both IFG and IGT (15-19%) as compared to those with isolated IFG (6-9%) or isolated IGT (4-6%) [44]. Our pre-diabetes patients were also at high risk for cardiovascular diseases as majority of them were obese with multiple co-morbidities including hypertension and dyslipidemia. In comparison to the data from National Health and Morbidity Survey 2011 [8], the lower level of physical activity observed in our study was likely due to the nature of our sample who were older, heavier, including greater proportions of females who had multiple co-morbidities. This finding was also consistent with other studies in which the risk of obesity among Malaysian adults was higher among females and among Malays and Indians compared to Chinese ethnic group [45,46].

The demographic profiles between our study and other studies in those diagnosed with pre-diabetes are comparable. For example, the study of physical activity and health-related quality of life in individuals with pre-diabetes by Taylor et al. [47] has reported their participants were mostly elderly (mean age 58 years compared to 52 years of our sample), females (73.3% compared to 64.2% of our sample) and had higher proportion of obese participants (58.6% compared to 71.3% of our sample). The study also found higher proportion of participants with multiple co-morbidities (100% compared to 88% of our sample) and similar mean BMI (31.2 ± 6.4 kg/m^2^ compared to 30.1 ± 4.8 kg/m^2^ of our study). The mean BMI in our sample was also found to be similar to those in Diabetes Prevention Program (DPP) in the Unites States [48] and the Finnish Diabetes Prevention Study (DPS) [49]. About half of our patients had a family history of diabetes. A cross sectional study among the South East Asian population showed that a positive family history was associated with increased risk of IFG or IGT (OR = 1.67, 95%CI = 1.42-1.97) and type 2 DM (OR = 2.95, 95% CI = 2.36-3.70) [50].

We found that the pre-diabetes patients showed lower physical component score as compared to mental health component score. This might imply that some of our pre-diabetes patients had difficulty in performing physical activities with bodily pain that reduced the range and extent of physical activity. This probably is due to the fact that more than one third of them had more than two co-morbidities including musculoskeletal diseases and majority were either overweight or obese. A relatively higher score in the mental component scores among our pre-diabetes patients showed that mental health was less affected by their pre-diabetes condition. Based on a qualitative study by Troughton et al. , some of the pre-diabetes individuals had issues of uncertainties about their diagnosis and its consequences, hence they considered pre-diabetes condition as less serious and easily accepted [51]. On the contrary, another population-based study comparing the HRQOL between the IFG and normal glucose tolerance people found significant lower mean scores demonstrated for physical functioning, bodily pain, physical component summary scores as well as mental component summary scores of the SF-36 among pre-diabetes individuals [17].

Pre-diabetes patients who were overweight and obese had significantly lower scores of physical component of SF-36 than those with normal weight after controlling for age, income, co- morbidities and physical activity level. Thus, this study affirmed this well recognized finding of previous study that higher BMI was associated with poorer PCS [52]. Similar findings were also found in other studies [53-55]. Another study among the Spanish population [18] found people with impaired glucose metabolism had lower PCS and MCS compared to people with normal glucose metabolism, especially among women.

The WHO BMI classification was used to identify those who were overweight and had obesity, however literature suggests that type 2 diabetes risk increased at a BMI cut off much lower than 25 kg/m^2^[27,30,56] as Asians had higher percentage of body fat compared to Caucasians at similar BMI [31,32,57]. Related to this, the mean BMI in this study was 30.1 kg/m2, with 21.6% overweight and 71.3% obese by the Asian classification [34]. However, there was not enough evidence to explain about the high prevalence of obesity among Malays in terms of genetic inheritance or susceptibility although Malays are phenotypically close to East Asians and Pacific Islanders. Based on available previous studies, high prevalence of obesity among Malays was mostly related to environmental, socio-cultural, dietary and physical activity factors [58].

Previous research has shown that physical activity is consistently associated with better HRQOL in the general population. People who regularly achieved the recommended level of physical activity reported better HRQOL than those who were physically inactive [59]. Furthermore, overweight and obese adults who met the recommended level of physical activity had higher levels of HRQOL than physically inactive adults [22]. However, physical activity was not a strong predictor of HRQOL in our study. Contrary to our expectations, few statistically significant mean differences in HRQOL outcomes were observed among overweight and obese individuals who were physically inactive, which is depicted in our figure. We believe the fact that the majority of patients in this study did not meet physical activity recommendations may have contributed to their low perceived HRQOL. Physical functional limitations due to other existed co-morbidities such as musculoskeletal diseases may have also influenced their poor quality of life. In addition, the small sample size in the subgroups of BMI may not have adequate power to produce significant associations between PA levels and HRQOL.

The results from this study provide important information about HRQOL of pre-diabetes patients and its association with BMI and physical activity level. There are, however, several limitations of this study that should be noted. This study comprised of a small and non-probability sample from the community, reducing the generalizability of the results.The cross sectional design of this study does not allow us to make any inferences about the causal associations between BMI, PA and HRQOL. Self reported physical activity may cause recall bias on the intensity of the physical activity. On the other hand, this may be the first study assessing the HRQOL among pre-diabetes patients in Malaysia. The direct uses of anthropometric measurements have provided us the BMI categories which were objectively measured. We also used the translated and validated questionnaire for assessing health-related quality of life.

## Conclusions

Pre-diabetes patients who were of normal weight reported higher HRQOL compared to those who were overweight and obese. These data suggest a potentially greater risk of poor HRQOL among pre-diabetes patients who are overweight and obese especially with regards to the physical health component. More research that examined the consequences of meeting the recommended level of physical activity among pre-diabetes is needed. These results support the rationale of a strategically designed intervention to improve their health and well being. Promoting recommended amount of physical activity and weight control are particularly important interventions for pre-diabetes patients at the primary care level.

## Abbreviations

HRQOL: Health-related quality of life; SF-36: Medical outcomes study short form 36-item health survey; IPAQ-: International physical activity questionnaire; BMI-: Body mass index; PA-Physical: Activity

## Competing interests

The authors declare that they have no competing interests.

## Authors’ contributions

All authors contributed to the conception and design of the study. NI and IA carried out data acquisition and management. NI and FMM carried out data analysis and drafted the manuscript. All authors read, commented and approved the final version of the manuscript.

## Pre-publication history

The pre-publication history for this paper can be accessed here:

http://www.biomedcentral.com/1471-2458/14/298/prepub
